# Prediction of Primary Dendrite Arm Spacing of the Inconel 718 Deposition Layer by Laser Cladding Based on a Multi-Scale Simulation

**DOI:** 10.3390/ma16093479

**Published:** 2023-04-29

**Authors:** Zhibo Jin, Xiangwei Kong, Liang Ma, Jun Dong, Xiaoting Li

**Affiliations:** School of Mechanical Engineering and Automation, Northeastern University, Shenyang 110819, China

**Keywords:** multi-scale simulation, primary dendrite arm spacing, phase field method, laser cladding, temperature gradient, nickel-based superalloy

## Abstract

Primary dendrite arm spacing (PDAS) is a crucial microstructural feature in nickel-based superalloys produced by laser cladding. In order to investigate the effects of process parameters on PDAS, a multi-scale model that integrates a 3D transient heat and mass transfer model with a quantitative phase-field model was proposed to simulate the dendritic growth behavior in the molten pool for laser cladding Inconel 718. The values of temperature gradient (*G*) and solidification rate (*R*) at the S/L interface of the molten pool under different process conditions were obtained by multi-scale simulation and used as input for the quantitative phase field model. The influence of process parameters on microstructure morphology in the deposition layer was analyzed. The result shows that the dendrite morphology is in good agreement with the experimental result under varying laser power (*P*) and scanning velocity (*V*). PDAS was found to be more sensitive to changes in laser scanning velocity, and as the scanning velocity decreased from 12 mm/s to 4 mm/s, the PDAS increased by 197% when the laser power was 1500 W. Furthermore, smaller PDAS can be achieved by combining higher scanning velocity with lower laser power.

## 1. Introduction

Nickel-based superalloys are widely used as a key material in aircraft structures due to their high fracture resistance and excellent corrosion resistance at a high temperature [1]. However, repairing or replacing damaged superalloy components using conventional production processes can be expensive. Laser cladding technology offers a cost-effective alternative for component repair or rapid manufacturing [2]. Laser cladding technology involves complex physical processes, including solid–liquid phase change, fluid flow and latent heat of melting, and is influenced by various process parameters such as laser power, scanning velocity, powder feeding rate and laser beam diameter. The choice of parameters significantly impacts the final microstructure morphology and mechanical properties [3]. Among them, primary dendrite arm spacing (PDAS) is an important parameter for characterizing microstructure, and can influence the performance of components, especially in laser processing. Due to the high temperature gradient and rapid cooling in laser cladding, the microstructure of the alloy produced by this process differs from that produced by conventional directional solidification. Additionally, PDAS size can effectively control microsegregation, which is directly related to the formation of delta phase in nickel-based superalloys. During both forging and cladding processes, excessive phase aggregation is a major cause of cracking [4,5]. Therefore, it is helpful to understand the relationship between process parameters and PDAS.

The microstructure of the laser cladding layer is a topic of ongoing interest in the field of laser cladding [6,7,8]. Numerous studies have shown that the microstructure and mechanical properties of laser cladding parts are primarily controlled by the real-time thermal characteristics of molten pool and the corresponding solidification parameters (temperature gradient *G* and solidification rate R) [9]. Microstructure characteristics can be effectively predicted by associating process parameters with the real-time thermal characteristics and solidification parameters [10]. Conventional theoretical methods estimate PDAS using solidification parameters: *λ_1_* = *AG^m^R^n^* [11], where *A* is the alloy constant and *m* and *n* are the model correlation indices. However, due to the complexity of the diffusion field around dendrites at high solidification rates, these approximate methods have large errors when applied to laser cladding [12].

Currently, high-temperature thermography and high-speed camera systems are used to monitor the solidification process of the molten pool [13,14,15] and measure the solidification parameters such as surface temperature and cooling rate. This allows analysis of the influence of solidification parameters on solidification structure size. However, on-site measurement of solidification conditions is challenging due to the high melt temperature and small size of the molten pool. Measured solidification parameters (such as temperature and fluid flow) are limited to the surface of the molten pool or thin melt layer, and experiment results are easily influenced by the working conditions [16,17,18].

Advances in computational ability and numerical methods have made computer simulation a popular tool for studying solidification problems. Many researchers have developed numerical models to analyze the evolution of solidification conditions in additive manufacturing processes and their impact on solidification microstructure characteristics [19,20]. The distribution of the macro-scale temperature field caused by the moving heat source is the key to determining solidification parameters. From this, solidification parameters such as the temperature gradient *G* and the solidification rate *R* at the S/L interface of the molten pool can be calculated. For micro-scale simulation, the cellular automata (CA) method and phase field (PF) method are commonly used to predict the solidification microstructure [21,22]. The phase-field method, in particular, is based on the thermodynamics of partial differential equations and explicitly tracks the interface, allowing the effective simulation of a complex S/L interface [23,24,25].

Currently, most multi-scale simulations of laser cladding use the finite element method with birth-death elements. However, this approach does not account for changes in the free surface or fluid flow in the molten pool and latent heat of melting. As a result, simulated solidification parameters may be inaccurate, leading to errors in predicting primary dendrite arm spacing in microscopic simulations [26].

The aim of this study is to link laser cladding process parameters with transient local thermal characteristics and solidification parameters to rapidly predict the solidification microstructure. Regarding Inconel 718 as the research object, a 3D transient laser cladding heat and mass transfer model was adopted to calculate the temperature gradient *G* and solidification rate *R* at the S/L interface of the laser cladding molten pool under different process parameters. Then, this was combined with a quantitative phase field model to further calculate the PDAS at the top of the molten pool. To validate the model, laser cladding experiments were carried out to analyze the integrated influence of process parameters on microstructure.

## 2. Mathematical Model

### 2.1. Macroscopic Heat and Mass Transfer Model

**Figure 1:**
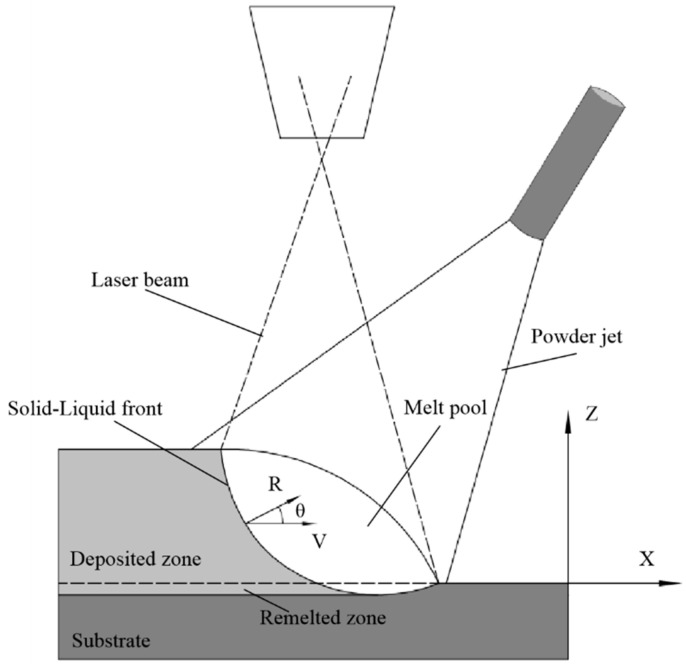
Schematic diagram of laser cladding.

During the laser cladding process, the laser beam moves continuously and the solidification front keeps pace with the heat source, as shown in Figure 1. The macroscopic temperature distribution caused by the moving heat source is the key to determining the solidification parameters. A 3D macroscopic heat and mass transfer model is established to simulate the temperature field, velocity field and molten pool morphology. The temperature gradient *G* and solidification rate *R* along the normal line of the S/L interface are further calculated. As the interface for the solid phase grows, each point at the solidification front grows in a direction perpendicular to the S/L interface at the corresponding *R* rate. *R* = *V*cos*θ*, where *θ* is the angle between the laser scanning direction and the normal solidification interface direction of the solidification interface in terms of molten pool geometry.

The simplified assumptions are as follows [27,28]:

(1) Liquid fluid flow in the molten pool is Newtonian, laminar and incompressible;
(2) The mushy region is assumed as a porous medium with isotropic permeability;
(3) Heat flux from heated powder and heat loss due to evaporation are neglected;
(4) The effect of shielding gas, powder feeding gas and powder on the surface of the molten pool is ignored.

The continuity equation is:

(1) $$\frac{\partial \rho }{\partial t}+\nabla \cdot \left(\rho u\right)=0$$

Due to the existence of S/L phase change in the laser cladding, the influence of latent heat on melting should be considered in the heat transport equation; the energy equation is [29]:

(2) $$\rho c_{p}\left(\frac{\partial T}{\partial t}+u\cdot \nabla T\right)=\nabla \cdot \left(k\nabla T\right)-\frac{\partial H}{\partial t}-\rho u\cdot \nabla H$$

In the above equation, the second and third terms on the right side represent unsteady terms of phase change enthalpy and the phase change enthalpy due to convection, respectively.

Where *ρ* is density, *c_p_* is specific heat, *T* is temperature, *t* is time, *k* is heat conduction dilution, ∆*H* is melting latent enthalpy capacity, and ∆*H* = *LF_l_*, *L* is latent heat of melting. The liquid mass fraction *F_l_* is defined as,

(3) $$F_{l}=\left\{\begin{array}{ll} 1, & T>T_{l}\\ \frac{T-T_{s}}{T_{l}-T_{s}}, & T_{s}<T\leq T_{{}_{l}}\\ 0, & T<T_{s} \end{array}\right.$$

The incompressible Navier–Stokes equation describes the liquid metal in the molten pool. Since velocity in the solid phase region is 0, a source term in the momentum equation is introduced to suppress velocity field in the solid phase region. In this study, the time source term in the liquid phase region is set to 0, leaving the momentum equation unchanged. In the solid phase region, however, the source term is infinite, and the remaining terms of the momentum equation become infinitesimally small. In the transition region, the equation degenerates to Darcy’s law relating porosity and pressure. Only pressure and source terms remain in the equation, while other terms become infinitesimally small.

(4) $$\rho \frac{\partial u}{\partial t}+\rho \left(u\cdot \nabla \right)u=\nabla \cdot (\mu \nabla u)-\frac{\partial p}{\partial x}+K_{0}\frac{\left(1-F_{l}\right)}{F_{l}{}^{3}+B}u$$

where ***μ*** is the viscosity, *p* is the pressure, and the third term on the right side of the Equation (4) represents the momentum dissipation term in the mushy region, which is based on the Carman–Kozeny Equation [30], *K*_0_ is a constant related to the mushy region, and *B* is a very small number to prevent the denominator from being zero. Linear interpolation is used to interpolate the thermophysical parameters of the mushy region.

The laser heat flow input at the liquid/gas interface is as follows:

(5) $$Q(x,y,t)=\frac{2P\eta_{l}}{\pi r_{l}{}^{2}}\exp(\frac{-2({\left(x-X(t)\right)}^{2}+{\left(y-Y(t)\right)}^{2})}{r_{l}{}^{2}})-h_{c}\left(T-T_{0}\right)-\sigma_{b}\varepsilon (T^{4}-T_{0}^{4})$$

where *P* is the laser power, *X*(*t*) and *Y*(*t*) are the *x* and *y* coordinate values of the laser center with time, *η_l_* is the absorption rate of the laser energy, *r_l_* is the radius of the laser source, *h_c_* is the heat transfer coefficient, *σ_b_* is the Stefan–Boltzmann constant, *ε* is the emissivity, and *T*_0_ is the ambient temperature.

The boundary condition of the momentum equation of the gas–liquid interface is [29],

(6) $$F_{L/G}=\sigma n^{*}\kappa -\nabla_{s}T\frac{d\sigma }{dT}$$

The two terms in Equation (6) represent capillary force and thermal capillary force, respectively. *σ* is the surface tension, n* is the surface normal, and *κ* is the surface curvature.

The description of the surface geometry of the molten pool is achieved through the moving mesh of the Lagrangian–Euler method (ALE). Two velocities are considered at the liquid/gas interface: fluid flow velocity and boundary movement velocity, caused by powder addition. They can be expressed as [31]:

(7) $$V_{L/G}=un^{*}-V_{p}\cdot n^{*}$$

where ***u*** is the flow velocity of the fluid of the liquid–gas interface, *V_p_* represents the moving velocity of the liquid–gas interface, and the calculation formula of *V_p_* is

(8) $$\;V_{p}\left(t,x\right)=\frac{2m_{f}\eta_{m}}{\rho_{m}\pi r_{p}^{2}}\exp\left(\frac{-2\left({\left(x-X\left(t\right)\right)}^{2}+{\left(y-Y(t)\right)}^{2}\right)}{r_{p}^{2}}\right)$$

In the above formula, *m_f_* is powder feeding rate, *η_m_* is powder capture rate, *ρ_m_* is powder density, *r_p_* is the radius of the laser source, *X*(*t*) and *Y*(*t*) are the moving track of the laser head with time, respectively.

### 2.2. Microscopic Phase-Field Model

The phase field model adopts the quantitative alloy phase field model proposed by Echebarria et al. [32], which is established under the thin interface limit to eliminate the dependence of the interface thickness, so as to describe the (non-conserved) phase field *ϕ* and (conserved) the solute field *c* during the solidification of dilute binary alloys. An inverse solute diffusion term is introduced into the model to avoid the solute diffusion effect at the diffusion interface at lower solidification rates. Inconel 718 is simplified as a Ni-Nb binary system containing only FCC γ phase and liquid phase with a mass fraction of 5% Nb. The scalar phase field parameter *ϕ* indicates whether a point in the two-dimensional field is liquid (*ϕ* = −1), solid (*ϕ* = 1), or a solid–liquid interface (−1 < *ϕ* < 1).

Ignoring the influence of latent heat effect, the freezing temperature approximation is adopted.

(9) *T* = *T_0_* + *G* (*z* − *Rt*)

where *T*_0_ (*z* = 0, *t* = 0) is a reference temperature, *G* is the temperature gradient along the Z direction, and *R* is the pulling velocity.

The governing equations of phase field and supersaturated concentration field in a two-dimensional system,

(10) $$\begin{array}{ll} \tau_{\phi }\left(\hat{n},z\right)\frac{\partial \phi }{\partial t} & =W_{0}^{2}\left\{\nabla \cdot \left[\alpha_{s}{\left(\hat{n}\right)}^{2}\nabla \phi \right]+\partial_{x}\left({\left|\nabla \phi \right|}^{2}\alpha_{s}\left(\hat{n}\right)\frac{\partial \alpha_{s}\left(\hat{n}\right)}{\partial \left(\partial_{x}\phi \right)}\right)+\partial_{z}\left({\left|\nabla \phi \right|}^{2}\alpha_{s}\left(\hat{n}\right)\frac{\partial \alpha_{s}\left(\hat{n}\right)}{\partial \left(\partial_{z}\phi \right)}\right)\right\}\\ & +\phi -\phi^{3}-\lambda {\left(1-\phi^{2}\right)}^{2}\left(U+\frac{z-Rt}{l_{T}}\right) \end{array}$$

(11) $$\left(\frac{1+k}{2}-\frac{1-k}{2}\phi \right)\frac{\partial U}{\partial t}=\nabla \cdot \left[D_{l}\frac{\left(1-\phi \right)}{2}\nabla U+{\vec{j}}_{at}\right]+\left[1+\left(1+k\right)U\right]\frac{1}{2}\frac{\partial \phi }{\partial t}$$

where $\tau_{\phi }\left(\hat{n},z\right)=\tau_{0}{\left(\alpha_{s}\left(\hat{n}\right)\right)}^{2}\left[1-\left(1-k\right)\frac{\left(z-Rt\right)}{l_{T}}\right]$, which is the temperature-dependent relaxation time. $\alpha_{s}\left(\hat{n}\right)=\left(1-3\delta \right)\left[1+\frac{4\delta }{1-3\delta }\left({\hat{n}}_{x}^{4}+{\hat{n}}_{z}^{4}\right)\right]$, which represents the two-dimensional fourfold anisotropy in the system, *δ* is strength of the surface tension anisotropy. $l_{T}=\frac{\left|m\right|c_{\infty }\left(1/k-1\right)}{G}$ is the thermal length, where *c*_∞_ ≡ *c* (*z* = +∞) is an initial alloy concentration (wt.%) far from the solidification front, *m* is liquid line coefficient, *k* is equilibrium partition coefficient, *D_l_* is the diffusivity of solute in liquid, $\hat{n}=\frac{\nabla \phi }{\left|\nabla \phi \right|}$, which is the unit vector normal to the interface. $U=\frac{1}{1-k}\left[\frac{ck/c_{\infty }}{\left(1-\phi \right)/2+k\left(1+\phi \right)/2}-1\right]$, generalized supersaturation field. ${\vec{j}}_{at}=\frac{1}{2\sqrt{2}}W_{0}\left[1+\left(1-k\right)U\right]\frac{\nabla \phi }{\left|\nabla \phi \right|}\frac{\partial \phi }{\partial t}$, anti-solute interception term. The anti-trapping current is given by Karma [33]. *W*_0_ is the value of setting interface thickness. *λ* is used as a parameter for the numerical convergence of the control model. *τ*_0_ stands for relaxation time. The interface thickness *W*_0_ = *d*_0_*λ*/*a*_1_ and the relaxation time *τ*_0_ = (*d*_0_^2^/*D*)*a*_2_*λ*^3^/*a*_1_^2^ are interrelated from a thin interface analysis, where *a*_1_ = 0.8839, *a*_2_ = 0.6267, and *λ* is treated as a parameter to control the numerical convergence of the model.

### 2.3. Model Parameters

The high-temperature physical properties of the Inconel 718 were calculated using J-mat pro and are presented in Table 1. A continuous laser with a radius of 1.1 mm was used. The powder mass flow rate is 10 g/min, with a capture efficiency of 0.9 and a flow density of 8.19 g/cm^3^.The powder flow radius was set to 2.3 mm. The values of these parameters are listed in Table 2.

The simulation model is a 10 mm × 20 mm × 5 mm cuboid with the cladding surface on the upper surface, serving as the input boundary for both the energy and mass transfer equations and the lifting surface for the ALE dynamic mesh. Mesh displacement is determined by the rise of the free surface due to powder addition and the influence of molten pool flow velocity on liquid surface advancement.

The phase field and concentration equations are solved on uniform meshes with a width of 50 µm in the *x* direction, and phase field parameters are listed in Table 3. The phase field governing equation *ϕ* is solved using a finite difference separation dispersion scheme, while the saturation concentration field U is solved using a finite-volume method with zero flux boundary conditions on both boundaries. The mesh spacing Δ*x* = Δ*y* = 0.005 µm, and the time step Δ*t* = 0.02*τ*_0_, while the value of *W*_0_ is about 10 times smaller than the dendrite tip radius calculated by the sharp interface model.

The maximum phase field interface thickness *W*_0_ = 0.01 µm, *λ* = 1.377 initializes the simulation along the bottom domain (at *y* = 0), and the S/L interface is randomly perturbed by small amplitude.

## 3. Experimental Materials and Scheme

Inconel 718 powder with a particle size of 45–105 µm and good flow characteristics was used in the experiment. The powder was dried at 100 ℃ for 2 h in a blast dryer prior to use. The elemental composition of powder elements is shown in Table 4:

A CO_2_ laser with a maximum power output of 4000 W was utilized in our experiment. Our team’s previous research indicated that laser power below 1500 W resulted in partial melting of the track due to powder deposition. Conversely, laser power above 2300 W caused powder vaporization and substrate melting due to splashing. Energy accumulation led to powder vaporization and substrate melting at scanning speeds below 4 mm/s. At higher scanning speeds (12 mm/s), partial melting of the trajectory was observed due to insufficient energy to melt the powder. So, laser power (1500 W, 1900 W, 2300 W) and scanning velocity (4 mm/s, 8 mm/s, and 12 mm/s) were selected for the orthogonal experiment, with a shielding gas flow rate of 10 L/min. The substrate was a 20 × 10 × 5 mm forging.

After wire cutting, surface oxide layers and stains were removed using an iron brush to prevent interference with cladding. Three cross sections perpendicular to the scanning direction were taken, and samples were ground and polished (using sandpaper grits of 240#, 600#, 800#, 1200#, and 2000# followed by polishing with W3.5 and W2.5 pastes). Samples were etched for 25 s using a solution of HCL, H_2_SO_4_ and CuSO_4_.Geometrical morphology was observed using an optical metallographic microscope and analyzed.

This study focused on dendrite growth in the deposited zone rather than the remelting zone. To ensure consistency in point positions across different process parameters, dendrite arm spacing was measured in an area below 0.2 mm from the top and near the centerline. The statistical method is to select three random-shaped ranges in the target area, and the area of the shape as *S*, and the number of dendrites is *N*. (Regions with less than 50% coverage at the boundary were marked as 0 and those with more than 50% coverage were marked as 1).

Three sections were taken from each sample and three positions were randomly selected within the target area for each cross-section. The cladding process was found to be quasi-steady-state, with *G* and *R* remaining unchanged for different x-coordinate values as long as y and z positions were consistent. Data from different cross-sections under the same process parameters were used to increase accuracy.

## 4. Results and Discussion

### 4.1. Solidification Characteristics of Molten Pool

**Figure 2:**
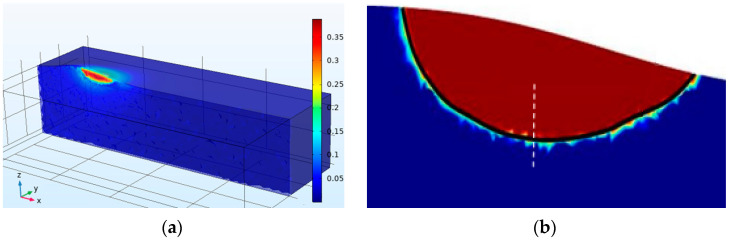
(**a**) The cross-section temperature field at 0.5 s, (**b**) Liquid phase temperature isotherm.

Nine cladding processes were simulated according to the orthogonal test. As energy is continuously input, the temperature in the molten pool increased until it exceeded the solidus temperature and a molten pool formed due to solid–liquid phase change. Figure 2a shows the temperature field at 0.5 s for a scanning velocity of *V* = 8 mm/s and laser power of *P* = 1900 W.

A quasi-steady-state is achieved after 0.5 s with an essentially unchanged isotherm shape. The back edge of the melt pool represented the solidification front and, after reaching quasi-steady-state, solidification parameters remained constant for all positions along the front. The longitudinal section of the molten pool (t = 1593 K) is taken as contour lines in Figure 2b, with the left part representing the solidification front.

**Figure 3:**
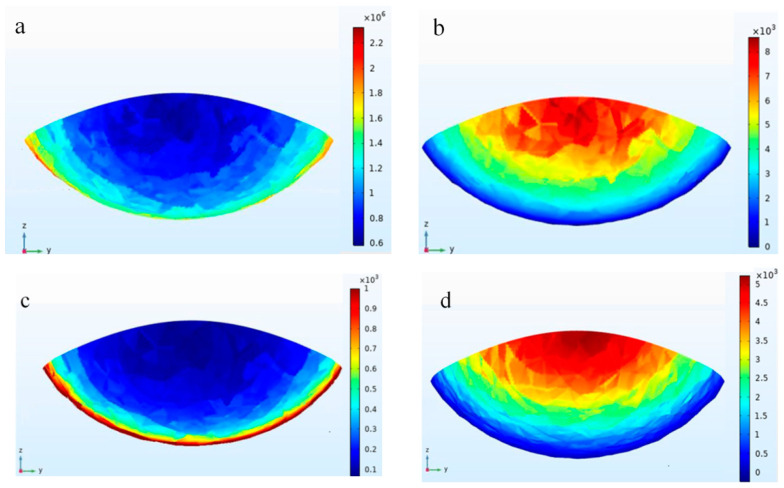
Computed contours of solidification characteristics: (**a**) temperature gradient *G*, (**b**) solidification growth rate *R*, (**c**) *G*/*R*, and (**d**) *G* × *R*.

As the laser source moves, a 3D solidification front surface also moves. Solidification parameters for all positions along the front are projected onto the cross-section of the deposition track to analyze molten pool characteristics (Figure 3).

As shown in Figure 3a, *G* increases from 1.6 × 103 K/m along the periphery to 2.2 × 103 K/m at the center of the cross-section, before decreasing sharply in the middle of the molten pool, and the temperature gradient *G* is smallest at the top of the pool.

The *R* value is shown in Figure 3b, and it is 8 mm/s at the top of the molten pool, which is the same as the scanning velocity, and decreases gradually to 0.2 mm/s at the surface of the deposited track.

Figure 3c,d show the contour of *G*/*R* and *G* × *R* over the projective plane, respectively. Higher *G*/*R* and lower *G* × *R* are observed at the bottom of the solidification front, while lower *G*/*R* and higher *G* × *R* are observed at the top. The *G*/*R* ratio governs the solidification mode, while *G* × *R* controls the scale of the solidification microstructure. In this study, microstructure scale is coarser at the bottom of the deposited track, and finer at the top. Solidification mode may transform from planar to cellular, columnar dendritic, and equiaxed dendritic as *G*/*R* decreases.

**Figure 4:**
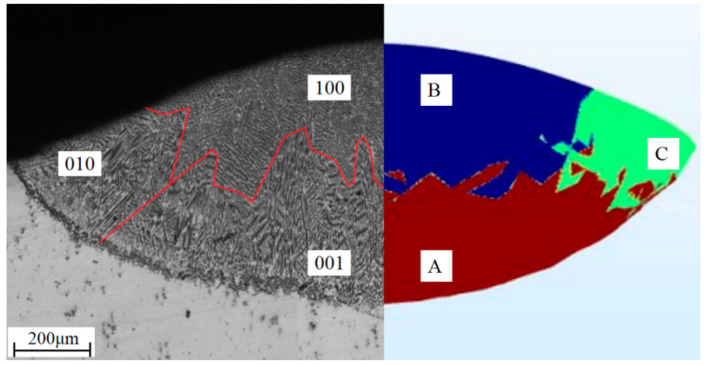
Comparison of dendrite growth direction between test and simulation; (A) *G*_[001]_ dominant region; (B) *G*_[100]_ dominant region; (C) *G*_[010]_ dominant region.

The values of *G* were calculated for each direction, and compared with the cladding layer obtained by the microstructure experimentally. As shown in Figure 4, *G*_[001]_ in the direction of [001] and *R*_[001]_ are dominant in region A, while *G*_[100]_ in the direction of [100] is dominant in region B, and *G*_[010]_ in the direction of [010] is dominant in region C.

The simulation results agree well with the experimental results. Columnar crystals grew vertically from the bottom of the molten pool and the dendrites deflected at positions where *G*_[001]_ is less than *G*_[100]_,forming a cross shape in the cross-section. 

**Figure 5:**
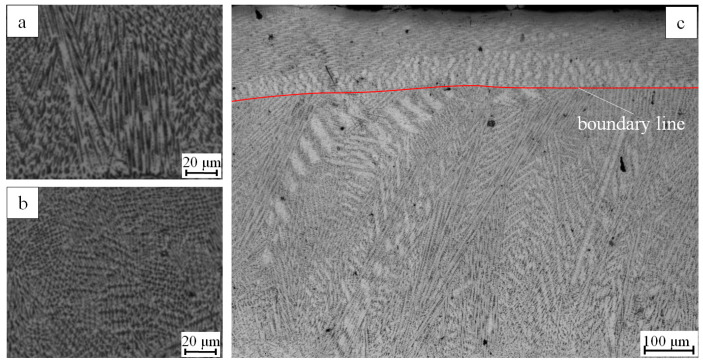
(**a**) Local enlarged drawing of Figure 4A; (**b**) Local enlarged drawing of Figure 4B; (**c**) the longitudinal section diagram.

Dendrite deflection is shown in Figure 5, with Figure 5a,b showing enlarged views of regions A and B in Figure 4, respectively, and Figure 5c showing a longitudinal section. A clear boundary line for dendrite growth direction can be observed in Figure 5c, with the dendrite growth direction at the top of the deposition area above the boundary differing from that at the bottom. Dendrites at the bottom of the deposition layer were thicker columnar crystals, while the microstructure at the top was more refined and smaller in size than dendrites at the bottom of the molten pool.

In this study, the microstructure of nine process parameters is considered, and *G* and *R* values are calculated as the comparative data in Table 5.

### 4.2. Simulation of Microstructure

**Figure 6:**
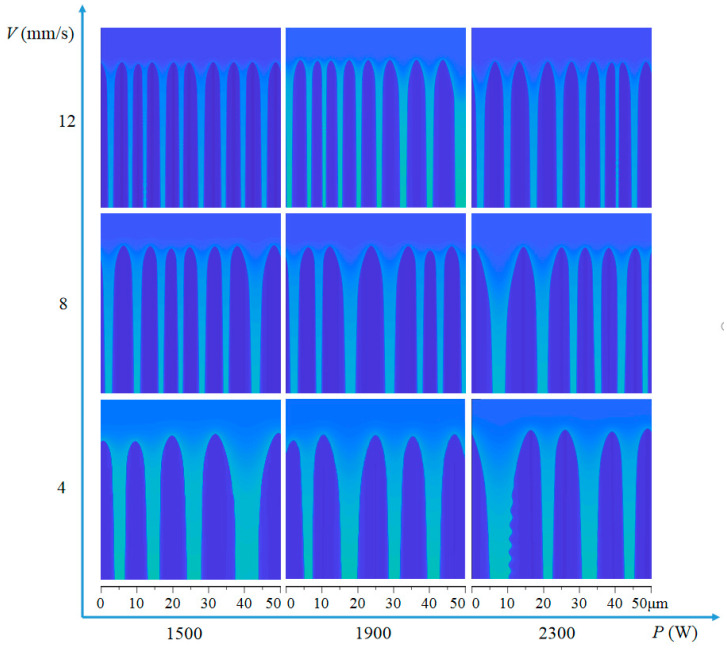
Steady-state cellular growth fronts for nine different solidification conditions.

*G* and *R* values for each process parameter were obtained and input into the phase field model. After the initial transients, Mullins–Sekerka instability rapidly developed at the solid–liquid interfaces, resulting in cellular structures that merged or split during growth. As solidification continue, the number of dendrites in the simulation region remain constant after a certain stage, with dendrite tips growing at a constant rate equal to the solidification rate, and average distance between neighboring γ-cells tips remaining constant. Steady-state dendrite morphology for various processes is shown in Figure 6. Mean PDAS is estimated from simulated cellular patterns by counting the number of cells along the system width (perpendicular to growth direction) and dividing by system width. The mean steady-state PDAS is between 5 µm to 12 µm in this case.

Figure 6 illustrates significant variations in Nb composition in the liquid ahead of tips, between cells, and across solid cells. Growing cells reject niobium into the liquid, enriching intercellular regions. Solidification of Inconel 718 consistently results in the formation of an Nb-rich phase. Related test results indicate that niobium is concentrated in the delta phase region, which is relatively brittle and prone to cracking [5]. Thus, simplification as a Ni-Nb binary alloy is plausible for describing major solidification behavior.

### 4.3. Influence of the Processing Parameters on the PDAS

**Figure 7:**
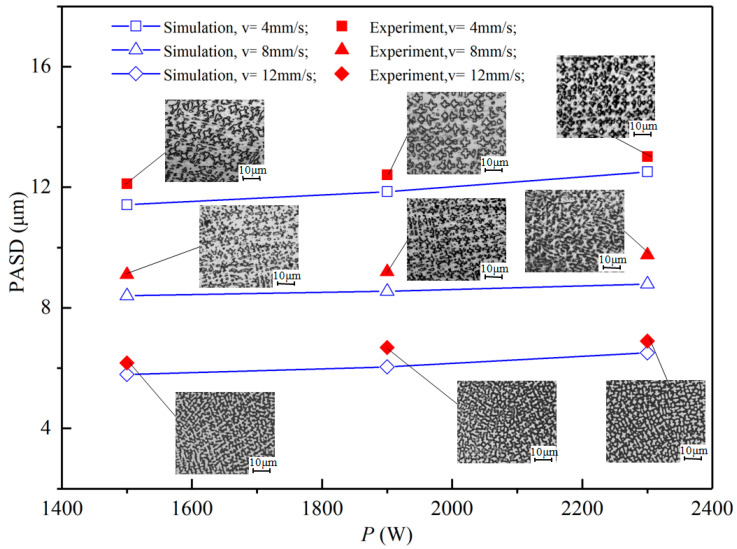
Variation trends of the PDAS with *P* at different *V*, OM image showing the transverse section of the laser track processed for nine different solidification conditions.

A comparison of the PDAS test and simulation results for Inconel 718 with nine process parameters is shown in Figure 7. Data comparison revealed a maximum error of 9.58% for process parameters 2300 W and 8 mm/s. Little difference is observed between PDAS values measured by simulation and experiment. The PF simulation was two-dimensional and solidification geometry may change slightly under solidification conditions in reality. However, the trend of simulation and experiment changing with scanning velocity and laser power was generally consistent, verifying model accuracy. Primary dendrite spacing decreased significantly with increasing laser scanning velocity and increased gently with increasing laser power.

To analyze the trend of PDAS with process parameters, it is necessary to reveal the relationship between solidification conditions and process parameters as PDAS is directly determined by specific alloy solidification conditions. Higher cooling rates resulted in smaller dendrite spacing, consistent with simulation results showing that *G* decreased with increasing *P* for a given *V*. This trend is similar to previous studies and is due to decreased heat flux from larger pools produced by higher *P*. The effect of process parameters on solidification rate *R* is more complex, with *R* increasing with increasing *P* at lower power and higher scanning velocity but *P* having no significant effect at higher scanning velocity. These comparisons show that the selection of characteristic solidification conditions primarily affects PDAS size but does not significantly change PDAS evolution trends. As long as suitable solidification characteristic conditions are selected, PDAS does not change during solidification.

**Figure 8:**
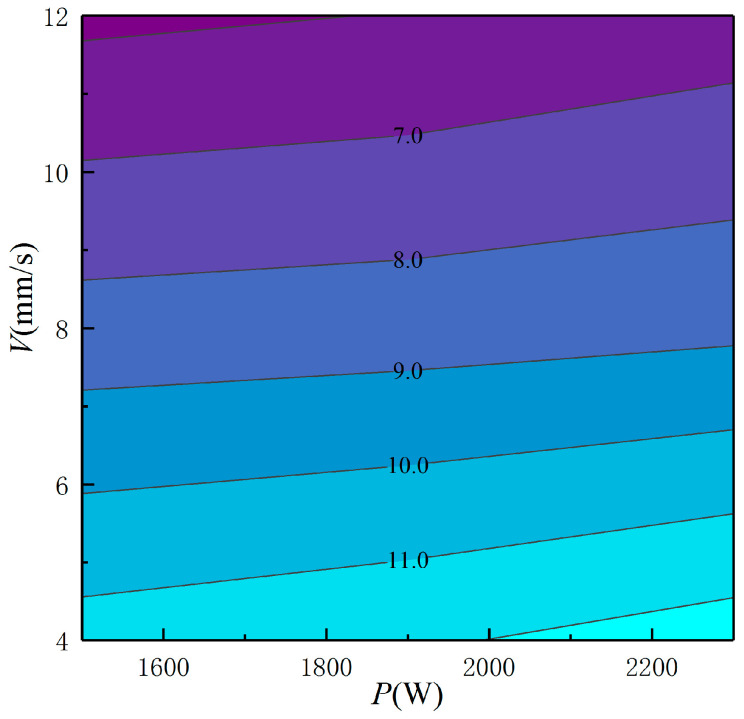
Combined effects of *P*, *V* on the PDAS (in μm).

It can be seen from Figure 8 that when laser power increases from 1500 W to 2300 W at a scanning velocity of 4 mm/s, PDAS increases from 5.79 μm to 6.51 μm. Increasing laser power increased molten pool energy input and heat accumulation, decreasing the molten pool cooling rate and leading to dendrite structure coarsening.

Scanning velocity (V) had a greater influence on PDAS than power. At constant power, PDAS increases significantly with decreased scanning velocity. At 1500 W power, increasing the scanning velocity from 12 mm/s to 4 mm/s increases PDAS by 197%. Scanning velocity changes affect both laser-molten pool interaction time and molten pool energy input. Increasing the scanning velocity decreases laser energy input and interaction time, reducing molten pool heat accumulation and increasing the substrate quenching effect. This significantly increases the molten pool cooling rate and refines the dendrite structure. Scanning speed is the most readily controllable process parameter and can be adjusted in real-time to achieve a desired microstructure.

It is generally accepted that low-energy input refines the solidification microstructure. Decreasing the laser power or increasing the scanning velocity directly decreases line energy input, increasing the molten pool cooling rate and refining the dendrite structure. Scanning velocity plays a leading role in the molten pool cooling rate and solidification structure, consistent with results reported by Muvvala [15].

## 5. Conclusions

In this study, a numerical model for predicting the PDAS of Inconel 718 laser cladding was established by combining a macroscopic transient 3D heat and mass transfer model with a quantitative phase field model. Solidification behavior in the molten pool under different process parameters was simulated, and the calculated results agreed well with the experimental values.

Simulated laser cladding showed that different positions within the cladding layer experienced different temperature histories and generated different dendrite morphologies. Finer equiaxed grains were obtained at the top of the cladding layer.

PDAS increased with increasing power and scanning velocity. Increased canning velocity resulted in an increase in monotonic PDAS with increasing power. Scanning velocity had a more pronounced effect on PDAS than power, with PDAS increasing significantly with increased scanning velocity. At 1500 W power, increasing the scanning speed from 12 mm/s to 4 mm/s increased PDAS by 197%. A finer microstructure could be obtained by combining a higher scanning speed with lower laser power.

The results showed that the multi-scale model provided better PDAS prediction for different processes, aiding the control and optimization of the laser cladding dendrite structure.

## Figures and Tables

**Table 1:** Physical parameters of Inconel 718.

Parameter	Value
Solidification spacing, Δ*T*	60 K
Melting point of pure nickel, *T_m_*	1726.15 K
Inconel 718 liquidus, *T_L_* (calculated by J-mat pro)	1635.14 K
Inconel 718 solidus, *T_S_* (calculated by J-mat pro)	1489.63 K
Fluid Diffusion Coefficient, *D* (calculated by J-mat pro)	0.7 × 10^−9^ m^2^/s
Gibbs-Thomson Coefficient, *Γ*	1.8 × 10^−7^ Km

**Table 2:** Macro simulation parameters.

Process Parameters	Value
Radius of beam, *r_l_*	1.1 mm
Powder feeding rate, *m_f_*	10 g/min
Powder flow radius, *r_p_*	2.3 mm
Powder capture efficiency, *η_m_*	0.9
Energy absorption efficiency of powder, *η_p_*	0.22
Convective Heat Transfer Coefficient, *h_c_*	10 W/(m^2^·K)

**Table 3:** Phase field parameters.

Parameter	Value
Initial alloy mass fraction, *c*_0_	5%
Equilibrium Partition Coefficient, *k_e_*	0.48
Liquidus Slope, *m_l_*	−10.5 K%^−1^
Equilibrium Freezing Range, *T*	57 K
Anisotropy Strength, *δ*	3%
Capillary Length, *d*_0_	8.0 × 10^−9^ m
Liquid Diffusion Coefficient, *D_l_*	3 × 10^−9^ m^2^ s^−1^
Solid Diffusion Coefficient, *D_s_*	10^−12^ m^2^ s^−1^

**Table 4:** Inconel 718 powder element content (wt.%).

Element	Ni	Co	Mo	Al	Nb	C	Cr
Content	52	0.1	2.94	0.53	5.16	0.037	18.7

**Table 5:** Nine process parameters and *G*, *R* values.

No.	*P* (W)	*V* (mm/s)	*G* (K/m)	*R* (m/s)
1	1500	4	4.42 × 10^5^	1.47 × 10^−3^
2	1500	8	6.09 × 10^5^	3.21 × 10^−3^
3	1500	12	6.48 × 10^5^	6.23 × 10^−3^
4	1900	4	4.65 × 10^5^	1.68 × 10^−3^
5	1900	8	5.41 × 10^5^	3.95 × 10^−3^
6	1900	12	5.69 × 10^5^	6.38 × 10^−3^
7	2300	4	4.76 × 10^5^	1.77 × 10^−3^
8	2300	8	4.85 × 10^5^	4.03 × 10^−3^
9	2300	12	4.98 × 10^5^	7.80 × 10^−3^
